# 直接进样-超高效液相色谱-三重四极杆质谱法测定水中31种全氟/多氟化合物

**DOI:** 10.3724/SP.J.1123.2025.09012

**Published:** 2026-05-08

**Authors:** Chenglong YANG, Pengfei LI, Yingying ZHANG, Lingjia WANG, Minmin HOU, Yali SHI, Yaqi CAI

**Affiliations:** 1.国科大杭州高等研究院环境学院，全省新污染物环境与健康重点实验室，浙江 杭州 310024; 1. School of Environment，Hangzhou Institute for Advanced Study，Zhejiang Key Laboratory of Environment and Health of New Pollutants，Hangzhou 310024，China; 2.中国科学院生态环境研究中心，环境化学与环境毒理全国重点实验室，北京 100085; 2. State Key Laboratory of Environmental Chemistry and Toxicology，Research Center for Eco-Environmental Science，Chinese Academy of Sciences，Beijing 100085，China; 3.中国科学院大学，北京 100049; 3. University of Chinese Academy of Sciences，Beijing 100049，China; 4.浙江省生态环境科学设计研究院，浙江 杭州 310007; 4. Ecological and Environmental Science and Research Institute of Zhejiang Province，Hangzhou 310007，China; 5.浙江省杭州生态环境监测中心，浙江 杭州 310012; 5. Hangzhou Ecological Environment Monitoring Center，Hangzhou 310012，China

**Keywords:** 全氟/多氟化合物, 直接进样, 超高效液相色谱-三重四极杆质谱法, 地下水, perfluoroalkyl and polyfluoroalkyl substances, direct injection, ultra performance liquid chromatography-triple quadrupole mass spectrometry, groundwater

## Abstract

本文建立了一种直接进样-超高效液相色谱-三重四极杆质谱测定水中31种全氟/多氟化合物（PFAS）的分析方法。通过对色谱柱、流动相及进样体积等参数的优化，最终确定以RSLC 120 C18色谱柱为分离柱，以甲醇和5 mmol/L乙酸铵水溶液为流动相进行梯度洗脱，在电喷雾负离子模式下，结合分时间段-多反应选择离子监测模式对31种PFAS进行定量分析。当样品进样体积为35 µL时，PFAS在其相应范围内具有良好的线性关系（*R*²> 0.994），检出限为0.007 1~3.0 ng/L，定量限为0.024~10 ng/L。加标水平为2、10和500 ng/L时，PFAS的回收率为67.2%~130.2%，相对标准偏差为0.30%~18%。应用该方法在10个地下水点位检出24种PFAS，总浓度（∑PFAS）范围为20.6~521 ng/L，其中PFOA和PFBA为主要污染物。该方法简便快捷，目标物覆盖范围广，准确度和灵敏度高，为环境水体中痕量PFAS的测定提供了高效可靠的方法选择。

## Abstract (en)

Perfluoroalkyl and polyfluoroalkyl substances （PFAS） possess desirable properties， including hydrophobicity， oleophobicity， surface activity， and thermal and chemical stability. Their extensive production and widespread application have resulted in the pervasive presence of PFAS in diverse environmental media. However， accumulating evidence indicates that PFAS are persistent， capable of long-range transport， bioaccumulative， and toxic； consequently， their adverse effects on ecosystems and humans are of widespread concern. Aquatic environments serve as a major transport pathway and contamination route for PFAS， making accurate measurement of PFAS levels in water crucial for assessing associated environmental and health risks. However， accurate quantification requires multi-step procedures， including sample filtration， enrichment， nitrogen blow-down concentration， and reconstitution， such as solid-phase extraction （SPE） and accelerated solvent extraction （ASE）. These methods are often labor-intensive and time-consuming. Although research on fully automated SPE technology is increasing， it necessitates installation of online SPE systems， which entail high costs and may present limitations in sample throughput per run. With continuous advancements in mass spectrometry， instrumental sensitivity has improved considerably， making direct injection of water samples for multi-analyte analysis technically feasible. However， reports on the use of direct injection methods for detecting PFAS in water remain limited， and the number of target analytes covered in such studies is relatively small. In this study， a direct injection-ultra performance liquid chromatography-triple quadrupole mass spectrometry （UPLC-MS/MS） method was developed for the determination of 31 PFAS in water. To optimize the chromatographic separation， enhance the detection sensitivity of target analytes， and minimize undesirable adsorption losses， the method was meticulously optimized with respect to solvent selection， injection volume， and syringe filter type. Our method involves the following procedure： 0.5 mL of water is aliquoted， mixed with 0.5 mL of methanol spiked with 2 ng of internal standard， and filtered through a 0.22 μm polypropylene membrane. The PFAS were analyzed by UPLC-MS/MS with an injection volume of 35 µL. The analytes were ionized in electrospray ionization negative mode （ESI^-^） with scheduled multiple-reaction monitoring （sMRM）. The MS parameters， including precursor and product ions， collision energy， and declustering voltage were optimized. Through optimization of the analytical column and mobile phases， the analytes were separated on an RSLC 120 C18 column with a gradient of methanol and 5 mmol/L ammonium acetate aqueous solution as the mobile phase in a gradient elution program. The results were quantified by the internal standard method. The method demonstrated excellent linearity （*R*²>0.994） across a defined concentration range. The limits of detection （LODs） and quantification （LOQs） were 0.007 1–3.0 ng/L and 0.024–10 ng/L， respectively. Recoveries at spiked levels of 2， 10， and 500 ng/L ranged from 67.2% to 130.2%， with relative standard deviations （RSDs） of 0.30% to 18%. To quantify the effective equivalence between the enrichment efficiency of SPE and the sensitivity of direct injection methods， a comparative analysis of analyte recovery rates was performed for both approaches. Furthermore， for long-chain PFAS， direct injection demonstrated consistent and favorable recovery performance. The method was applied to analyze PFAS in groundwater samples. The results showed that 24 PFAS were detectable with the total PFAS content （∑PFAS） ranging from 20.6 to 521 ng/L， with perfluorooctanoic acid （PFOA） and perfluorobutanoic acid （PFBA） being the primary pollutants. This approach is simple， rapid， highly sensitive， and provides broad coverage of target analytes， making it suitable for the quantitative analysis of PFAS in urban groundwater. It offers an efficient and reliable technical solution for determining trace-level PFAS in environmental water samples.

全氟/多氟化合物（perfluoroalkyl and polyfluoroalkyl substances， PFAS）是一类分子结构中与碳原子相连的H全部或部分被F所取代的人工合成化学品，具有理想的疏水疏油、表面活性和热化学稳定等特性，广泛应用于生产及生活消费的各个领域^［1］^。截至2020年，全球市场已有超过4 700种PFAS被用于200多个不同的领域^［2］^。它们的生产和使用导致PFAS广泛存在于各种环境介质中，目前已在土壤^［3］^、地表水^［4］^、地下水^［5］^和灰尘^［6］^等介质中检出多种PFAS。存在于环境中的PFAS主要包括全氟烷基羧酸类（PFCAs）、全氟烷基磺酸类（PFSAs）以及全氟调聚醇（FTOHs）等，其中针对PFCAs和PFSAs的研究最为深入，毒理学研究表明全氟辛酸（PFOA）和全氟辛基磺酸（PFOS）会对人体肝脏和免疫系统功能产生不利影响^［7，8］^。从21世纪开始其环境风险引起了广泛关注，PFOS和PFOA及其相关物质分别于2009年和2019年被列入《斯德哥尔摩公约》。2023年我国颁布实施的《重点管控新污染物清单（2023版）》也包含有这两种物质^［9］^。严格的市场监管促使短碳链（C≤7）或多氟类PFAS作为替代品开始生产和使用^［10］^。然而，目前研究显示某些替代品对生物体的神经、发育等仍具有较高的毒性效应，由此引起的环境污染和健康风险不容忽视^［11，12］^。由于大部分PFAS具有较强的极性和较高的水溶性，易分布于环境水体，因此水环境成为PFAS重要的污染和传输媒介^［13，14］^，其在各种水体中的污染水平研究引起了高度重视。

水环境中PFAS的含量一般处于ng/L~µg/L级别，美国环境保护署（EPA）最新发布的《国家生活饮用水标准》（National Primary Drinking Water Regulations， NPDWR）规定PFOA和PFOS的最大允许含量均为0.004 µg/L^［15］^；我国《生活饮用水卫生标准》（GB 5749-2022）^［16］^也将PFOA和PFOS作为参考指标纳入其中，规定其最大允许含量分别为0.08 µg/L和0.04 µg/L。目前，水中PFAS的常用检测方法大多采用液相色谱-串联质谱法（LC-MS/MS）。对于痕量PFAS检测，常用的前处理方法包括液液萃取、索氏提取、固相萃取、加速溶剂萃取、超声萃取以及衍生化技术等^［17］^。美国EPA于2024年发布并实施的固相萃取-LC-MS/MS标准分析方法（Method 1633）^［18］^，是针对水体中PFAS检测较为全面的技术标准方法。我国现有的标准方法如GB/T 5750.8-2023《生活饮用水标准检验方法 第8部分：有机物指标》^［19］^和HJ 1333-2023《水质 全氟辛基磺酸和全氟辛酸及其盐类的测定 同位素稀释/液相色谱-三重四极杆质谱法》^［20］^也都选用弱阴离子交换固相萃取柱对PFAS进行富集净化^［21］^，方法的样品浓缩倍数为200~1 000，方法检出限可达ng/L，甚至pg/L级别。但以上方法均需要进行样品过滤、富集、氮吹浓缩和定容等多个步骤^［22］^，存在操作烦琐、成本高、分析目标物数量有限等问题。近年来，全自动固相萃取技术的研究逐渐增多^［23，24］^，但需要加装在线固相萃取装置，成本高，耗时长，单次可处理样品数量有限。随着质谱技术的不断发展，仪器灵敏度呈现量级式的提高，技术上具备了水样直接进样进行多目标物分析的条件。直接进样法仅需过滤即可进样，在简化操作、提高效率和降低成本方面具有显著优势，近年来在水样中PFAS的快速分析中得到应用^［25］^。吴宇峰等^［26］^开发了直接进样法检测地表水中的12种PFAS，进样量20 µL，方法检出限20~50 ng/L；吉义平等^［27］^则将该技术应用于污水处理厂出口水，分析了18种PFAS，进样量50 µL，检出限0.7~7.8 ng/L。然而，直接进样法由于未对水中PFAS进行浓缩富集，因此其灵敏度仍显不足，且对宽谱系PFAS的覆盖度不够。目前面临的核心挑战是如何在不依赖样品浓缩的前提下，凭借仪器自身性能优势和方法优化实现对复杂环境基质中目标物的精准定量。因此，围绕UPLC-MS/MS开发短中链及新型PFAS的直接进样快速分析方法，不仅是对现有分析方法体系的有益补充，而且有助于环境水体中PFAS的快速精准监测，具有重要的现实意义。

本文针对上述PFAS分析方法在目标物覆盖范围与检测灵敏度方面的不足，以地下水为代表性基质，系统优化了色谱柱类型、流动相组成及进样体积等参数，建立了一种直接进样-UPLC-MS/MS分析方法，实现了地下水中31种PFAS（包括短链、长链及新型替代物）的同时检测。

## 1 实验部分

### 1.1 仪器、试剂与材料

LC-40超高效液相色谱仪（日本Shimadzu公司）及Triple quad^TM^ 7500三重四极杆质谱仪（美国AB SCIEX 公司）；CNW24位固相萃取真空装置（德国CNW公司）；ND200-2氮气吹扫仪（杭州瑞诚仪器有限公司）。

甲醇、乙腈和乙酸铵均为色谱纯，购自美国ThermoFisher公司；冰醋酸（优级纯，>99.8%）和氨水（优级纯）购自国药集团化学试剂有限公司；实验用水为蒸馏水，购自屈臣氏有限公司；针式过滤器（0.22 µm，13 mm）购自中国津腾实验设备有限公司；Oasis WAX 固相萃取柱（6 mL，150 mg，60 µm）购自美国Waters公司；玻璃纤维滤膜（0.7 µm，47 mm i.d.）购自英国Whatman公司。31种PFAS标准品（>98%）以及9种同位素标记物（见表1），除全氟壬烯氧基苯磺酸（OBS）为凯尔氟新材料有限公司的商业产品（95%，工业级）纯化而来，其余均购自加拿大Wellington公司。

**表 1:** 31种PFAS和9种内标的保留时间和质谱参数

No.	Compound	Chinese name	RT/min	Precursor ion （*m/z*）	Product ion （*m/z*）	EP/V	CE/eV	CXP/V	Q0D/V	IS
	**Perfluoroalkyl carboxylic acids （PFCAs， 全氟烷基羧酸）**									
1	perfluorobutanoic acid （PFBA）	全氟丁酸	5.70	212.9	168.9^*^	-5.0	-12.5	-10.0	-20.0	^13^C_4_-PFBA
2	perfluoropentanoic acid （PFPeA）	全氟戊酸	6.09	263.0	218.9^*^	-10.0	-11.7	-11.0	-9.0	^13^C_4_-PFBA
3	perfluorohexanoic acid （PFHxA）	全氟己酸	6.45	312.9	269.0^*^	-13.0	-14.2	-9.5	-14.0	^13^C_2_-PFHxA
4	perfluoroheptanoic acid （PFHpA）	全氟庚酸	6.87	362.9	318.9^*^	-10.0	-12.0	-16.0	-16.5	^13^C_4_-PFOA
5	perfluorooctanoic acid （PFOA）	全氟辛酸	7.35	412.9	168.9^*^	-10.0	-22.9	-8.0	-15.0	^13^C_4_-PFOA
			7.35		368.9	-10.0	-12.4	-15.0	-15.0	
6	perfluorononanoic acid （PFNA）	全氟壬酸	7.99	462.9	419.0^*^	-10.0	-13.8	-17.5	-15.0	^13^C_5_-PFNA
7	perfluorodecanoic acid （PFDA）	全氟癸酸	8.37	512.9	469.0^*^	-10.0	-15.8	-17.0	-20.0	^13^C_2_-PFDA
8	perfluoroundecanoic acid （PFUnDA）	全氟十一酸	8.88	563.0	518.9^*^	-10.0	-16.0	-20.0	-20.0	^13^C_2_-PFUnDA
9	perfluorododecanoic acid （PFDoDA）	全氟十二酸	9.42	613.0	569.0^*^	-11.0	-17.0	-10.5	-10.8	^13^C_2_-PFDoDA
10	perfluorotridecanoic acid （PFTrDA）	全氟十三酸	9.98	662.9	618.9^*^	-12.1	-18.0	-9.0	-20.0	^13^C_2_-PFDoDA
11	perfluorotetradecanoic acid （PFTeDA）	全氟十四酸	10.51	712.8	669.0^*^	-6.5	-20.1	-16.2	-20.0	^13^C_2_-PFDoDA
	**Perfluoroalkane sulfonic acids （PFSAs， 全氟烷基磺酸）**									
12	perfluorobutane sulfonic acid （PFBS）	全氟丁基磺酸	6.11	298.8	79.9^*^	-6.0	-62.0	-8.5	-20.0	^18^O_2_-PFHxS
			6.21		99.0	-10.0	-34.8	-7.0	-20.0	
13	perfluoropentane sulfonic acid （PFPeS）	全氟戊基磺酸	6.44	348.8	79.7^*^	-4.0	-70.0	-7.0	-10.1	^18^O_2_-PFHxS
			6.44		98.9	-3.0	-43.5	-14.0	-30.3	
14	perfluorohexane sulfonic acid （PFHxS）	全氟己基磺酸	6.97	398.8	79.9^*^	-10.0	-84.0	-9.0	-20.0	^18^O_2_-PFHxS
			6.84		99.0	-10.0	-75.0	-6.0	-20.0	
15	perfluoroheptane sulfonic acid （PFHpS）	全氟庚基磺酸	7.32	448.9	79.9^*^	-10.0	-86.0	-15.0	-10.0	^18^O_2_-PFHxS
			7.44		98.9	-7.0	-80.0	-7.0	-10.0	
16	perfluorooctane sulfonic acid （PFOS）	全氟辛基磺酸	7.83	498.9	79.9^*^	-10.0	-110.0	-9.0	-20.0	^13^C_4_-PFOS
			7.94		99.0	-10.0	-91.0	-9.0	-30.0	
17	perfluorononane sulfonic acid （PFNS）	全氟壬基磺酸	8.46	548.8	79.8^*^	-5.5	-112.5	-7.3	-20.0	^13^C_4_-PFOS
			8.33		98.8	-7.5	-89.3	-9.5	-10.0	
18	perfluorodecane sulfonic acid （PFDS）	全氟癸基磺酸	8.97	598.8	79.9^*^	-11.0	-126.2	-9.2.0	-7.2	^13^C_4_-PFOS
			8.97		99.0	-12.5	-107.4	-12.1	-20.0	
	**Other PFASs （其他PFASs）**									
19	6∶2 chlorinated polyfluoroalkyl ether sulfonic acid （6∶2 Cl-PFESA）	6∶2氯代多氟醚磺酸	8.09	530.6	351.0^*^	-10.0	-34.6	-12.0	-20.0	^13^C_4_-PFOS
			8.09		83.0	-6.0	-58.0	-4.0	-20.0	
20	8∶2 chlorinated polyfluoroalkyl ether sulfonic acid （8∶2Cl-PFESA）	8∶2氯代多氟醚磺酸	9.24	630.7	450.9^*^	-10.0	-38.5	-21.8	-20.0	^13^C_4_-PFOS
			9.09		82.9	-10.0	-72.0	-10.0	-21.0	
21	10∶2 chlorinated polyfluoroalkyl ether sulfonic acid （10∶2Cl-PFESA）	10∶2氯代多氟醚磺酸	10.18	730.7	550.9^*^	-11.0	-45.0	-16.0	-21.0	^13^C_4_-PFOS
			10.34		82.9	-7.8	-100.0	-9.8	-20.0	
22	perfluorononenoxybenzenesulfonic acid （OBS）	全氟壬烯氧基苯磺酸	8.76	602.8	108.2	-11.4	-100.0	-5.3	-20.0	^13^C_4_-PFOS
			8.62		171.9^*^	-9.1	-50.7	-10.0	-21.0	
23	perfluorobutane sulfonamide （FBSA）	全氟丁基磺酰胺	6.86	297.8	77.9^*^	-10.0	-50.0	-13.0	-20.0	^18^O_2_-PFHxS
24	perfluorohexane sulfonamide （FHxSA）	全氟己基磺酰胺	8	397.9	77.9^*^	-14.0	-61.0	-8.5	-20.0	^18^O_2_-PFHxS
25	perfluorooctane sulfonamide （FOSA）	全氟辛基磺酰胺	9.19	497.9	77.9^*^	-13.0	-83.8	-10.0	-14.0	^13^C_4_-PFOS
26	*N*-methyl perfluorooctane sulfonamide （*N*-MeFOSAA）	*N*-甲基全氟-1-辛烷磺酰胺基乙酸	8.80	569.9	419.0^*^	-11.5	-28.5	-14.5	-20.0	^13^C_2_-PFDA
			8.80		512.0	-6.7	-28.8	-20.3	-20.0	
27	*N*-ethyl perfluorooctane sulfonamido acetic acid （*N*-EtFOSAA）	*N*-乙基全氟-1-辛烷磺酰胺基乙酸	8.93	584.0	419.0^*^	-3.0	-27.5	-15.0	-9.7	^13^C_2_-PFDA
28	sodium 1*H，*1*H，*2*H，*2*H*-perfluorohexane sulfonic acid （4∶2 FTS）	4∶2氟调聚物磺酸	6.42	326.8	81.0	-4.2	-52.7	-9.9	-20.0	^18^O_2_-PFHxS
			6.55		307.0^*^	-2.5	-28.0	-13.5	-13.5	
29	sodium 1*H，*1*H，*2*H，*2*H*-perfluorooctane sulfonic acid （6∶2 FTS）	6∶2氟调聚物磺酸	7.48	426.7	81.0	-11.4	-73.5	-9.0	-20.0	^13^C_4_-PFOS
			7.46		407.0^*^	-12.0	-31.5	-25.6	-20.0	
30	sodium 1*H，*1*H，*2*H，*2*H*-perfluorodecane sulfonic acid （8∶2FTS）	8∶2氟调聚物磺酸	8.52	526.5	81.0	-13.7	-83.2	-14.2	-14.0	^13^C_4_-PFOA
			8.52		506.9^*^	-8.5	-38.4	-9.8	-22.0	
31	hexafluoropropylene oxide dimer acid （HFPO-DA）	六氟环氧丙基二聚体	6.54	328.9	284.9^*^	-10.0	-9.0	-8.0	-20.0	^13^C_4_-PFOA
	**ISs**									
32	perfluoro-*n*-（1，2，3，4-^13^C_4_）butanoic acid （^13^C_4_-PFBA）	^13^C_4_-全氟丁酸	5.79	216.9	171.8	-10.0	-12.5	-13.0	-20.0	
33	perfluoro-*n*-（1，2-^13^C_2_）hexanoic acid （^13^C_2_-PFHxA）	^13^C_2_-全氟己酸	6.57	315.0	270.0	-6.0	-12.5	-9.5	-20.0	
34	perfluoro-*n*-（1，2，3，4-^13^C_4_）octanoic acid （^13^C_4_-PFOA）	^13^C_4_-全氟辛酸	7.46	417.0	372.0	-13.0	-14.1	-15.0	-14.0	
35	perfluoro-*n*-（1，2，3，4，5-^13^C_5_）nonanoic acid （^13^C_5_-PFNA）	^13^C_5_-全氟壬酸	7.98	468.0	423.2	-10.0	-15.1	-17.5	-10.0	
36	perfluoro-*n*-（1，2-^13^C_2_）decanoic acid （^13^C_2_-PFDA）	^13^C_2_-全氟癸酸	8.49	514.9	470.0	-10.0	-15.0	-15.0	-15.0	
37	perfluoro-*n*-（1，2-^13^C_2_）dodecanoic acid （^13^C_2_-PFDoDA）	^13^C_2_-全氟十二酸	9.58	614.7	570.0	-3.0	-17.6	-13.0	-20.0	
38	perfluoro-*n*-（1，2-^13^C_2_）undecanoic acid （^13^C_2_-PFUnDA）	^13^C_2_-全氟十一酸	9.02	565.0	520.2	-8.0	-16.8	-20.0	-20.0	
39	perfluoro-1-hexane（¹⁸O₂）sulfonic acid （^18^O_2_-PFHxS）	^18^O_2_-全氟己烷磺酸	6.97	402.9	103.0	-10.0	-75.0	-10.0	-20.0	
40	perfluoro-*n*-（1，2，3，4-^13^C_4_）octane sulfonic acid （^13^C_4_-PFOS）	^13^C_4_-全氟辛烷磺酸	7.93	503.0	79.9	-4.0	-108.0	-9.0	-20.0	

RT： retention time； EP： entrance potential； CE： collision energy； CXP： collision cell exit potential； Q0D： Q0 dissociation； * quantitative ion.

### 1.2 标准溶液配制

分别准确量取31种PFAS标准品（包括30种混合标准溶液与1种OBS单标）及9种同位素内标，以甲醇为溶剂进行稀释，配制成质量浓度分别为1 µg/mL与0.1 µg/mL的储备液，于-20 ℃条件下避光保存。

使用前，取适量储备液，以甲醇逐级稀释，制备得到100 ng/mL的混合标准使用液与40 ng/mL的混合同位素内标使用液。精确移取0.5 mL纯水，依次加入不同体积的混合标准使用液及50 µL混合同位素内标使用液，随后补加甲醇至总体积为1.0 mL，涡旋混匀，配制校准曲线工作溶液。

### 1.3 样品前处理

取0.5 mL水样于离心管中，加入50 µL 40 ng/mL的混合同位素内标使用液，再加入甲醇使总体积为1.0 mL，涡旋混匀，过0.22 μm聚丙烯滤膜（不经任何预冲洗步骤），待检测。

### 1.4 仪器检测条件

#### 1.4.1 色谱条件

色谱柱：Acclaim RSLC 120 C18 （150 mm×2.1 mm，2.2 µm，美国ThermoFisher公司）作为分析柱，Hypersil Gold C18 （50 mm×4.6 mm，1.9 µm，美国ThermoFisher公司）作为延迟柱串联在流动相混合器和进样器之间，以消除液相色谱系统本身的背景干扰；流动相：A为5 mmol/L乙酸铵水溶液，B为甲醇；柱温35 ℃；流速0.3 mL/min；进样量35 µL。梯度洗脱程序：0~1 min，5%B；1~2 min，5%B~70%B；2~9 min，70%B~90%B；9~12 min，95%B；12~12.1 min，95%B~5%B；12.1~15 min，5%B。

#### 1.4.2 质谱条件

采用电喷雾离子源（ESI），负离子模式检测。喷雾电压：-2 000 V；气帘气（CUR）压力：310 kPa；雾化气（GS 1）压力：241 kPa；加热辅助气（GS 2）压力：482 kPa；离子源温度（TEM）：350 ℃。采用分段多反应监测（scheduled multiple reaction monitoring， sMRM）模式采集数据。31种PFAS和9种内标的质谱分析参数如表1所示。

## 2 结果与讨论

### 2.1 色谱和质谱条件的优化

#### 2.1.1 质谱参数优化

在ESI⁻模式下，将31种目标PFAS的混合标准溶液（1 000 ng/L）经针泵进样直接导入质谱系统，通过全扫描及子离子扫描确定各化合物的最佳质谱参数，包括：母离子/特征子离子对、入口电压（EP）、碰撞能量（CE）及碰撞电压（CXP）。数据采集采用sMRM模式。相较于常规的多反应监测模式（MRM），sMRM基于目标物的保留时间窗口（±40 s），在特定时间段监测该洗脱物离子对，可显著增加离子的驻留时间。该策略可有效规避全时间周期、全通道扫描导致的信号稀释，显著提升目标物响应强度及信噪比（*S/N*）。优化后的质谱参数详见表1。

#### 2.1.2 色谱柱优化

本研究选定的目标PFAS种类多，极性分布范围较宽，为优化并兼顾各目标物的色谱保留行为，考察了ACQUITY UPLC BEH C18 （100 mm×2.1 mm， 1.7 µm，美国Waters公司）和Acclaim RSLC 120 C18 （150 mm×2.1 mm，2.2 µm）两种C18色谱柱对31种PFAS的分离效果和保留能力。结果表明，两者在峰面积值上无明显差异。但观察到BEH C18色谱柱的色谱峰对称性及柱效相对较差，有明显的峰拖尾现象（见图1a）。综合考量分离性能与分析灵敏度，最终选择RSLC 120 C18色谱柱作为后续分析的分离色谱柱。

**图1:**
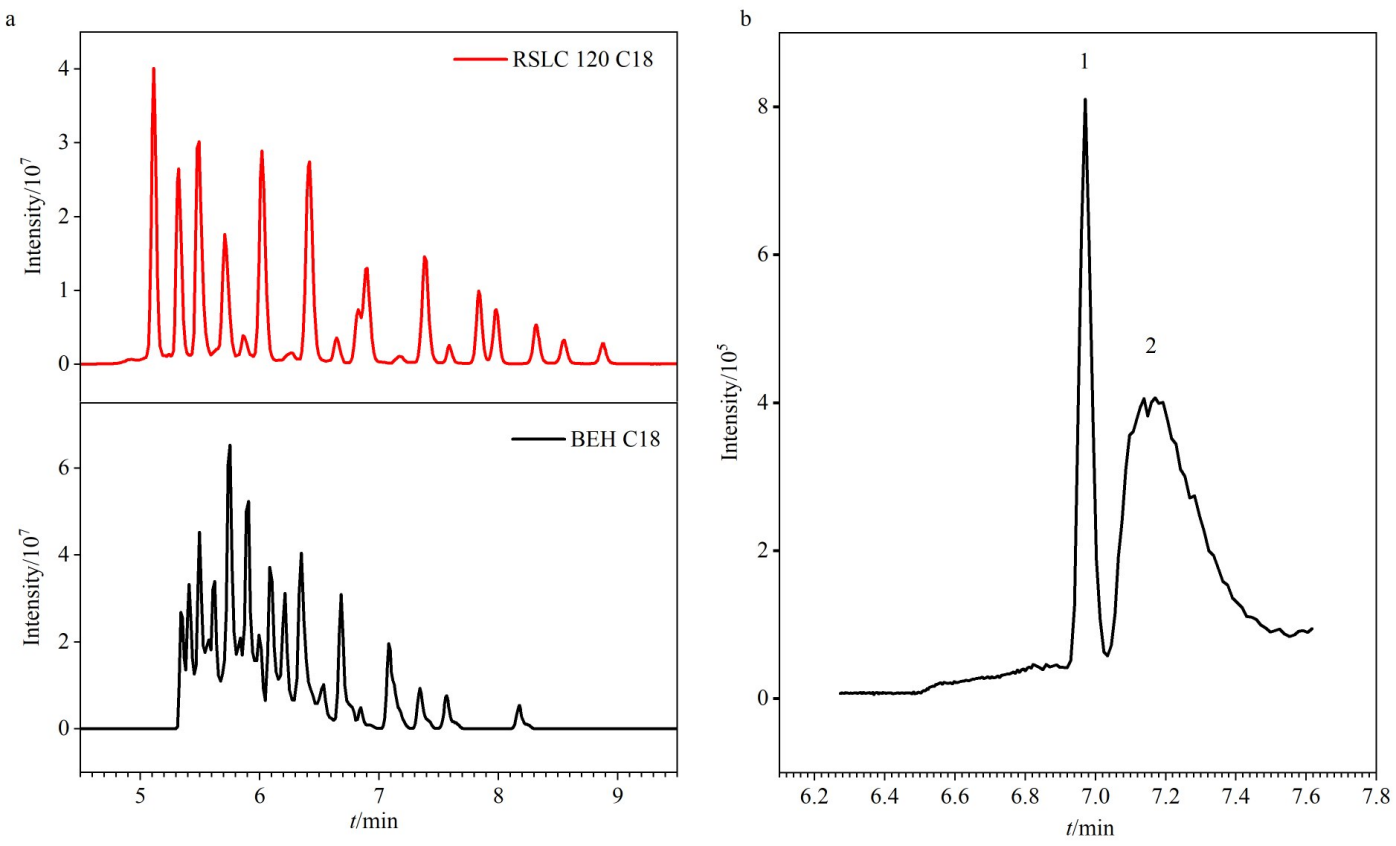
（a）采用不同色谱柱时31种PFAS的总离子流色谱图和（b）延迟柱对PFBA与系统干扰物的分离效果

为使来自流动相和UPLC泵系统的污染降至最低，在泵的流动相混合阀和自动进样器进样阀之间连接延迟柱Hypersil Gold C18，以分离来自流动相和UPLC泵系统中潜在的PFAS。分析物峰可以通过延迟柱与系统污染峰分开（见图1b）。该措施有效避免了背景PFBA的干扰，提升了方法的检测可靠性。

#### 2.1.3 流动相优化

目标PFAS既具有疏水的烷基链，又有亲水的官能团，多数研究采用甲醇-甲酸铵或乙腈-乙酸铵等流动相体系进行梯度洗脱^［23，24，28］^。为了提高色谱分离效果和目标物的检测灵敏度并获得良好峰形，本研究系统对比了甲醇-水与乙腈-水两种流动相体系对PFAS分离效果和灵敏度的影响。结果表明：除部分长链PFCAs（C≥8）在甲醇-水体系中峰面积值较低外，其余多数PFAS的峰面积值均高于乙腈-水体系。同时，在乙腈-水体系中观察到多处峰分叉现象，整体分离效果差。有研究报道，ESI⁻模式下，在水相中添加挥发性缓冲盐（如乙酸铵）可促进目标物形成稳定去质子化离子（［M-H］⁻），提升离子化效率，实现更好的分离度和更高的灵敏度^［29］^。为确定最佳添加剂浓度，考察0、2、5、8及10 mmol/L乙酸铵溶液对目标物响应值和分离度的影响。结果表明，目标物峰面积并未显著提升，但保留时间随着乙酸铵浓度（2~10 mmol/L）的增加而增加。同时，如图2所示，乙酸铵浓度为5 mmol/L时色谱峰形尖锐且对称，整体分离度良好。最终选择甲醇-5 mmol/L乙酸铵水溶液为最佳流动相体系。

**图2:**
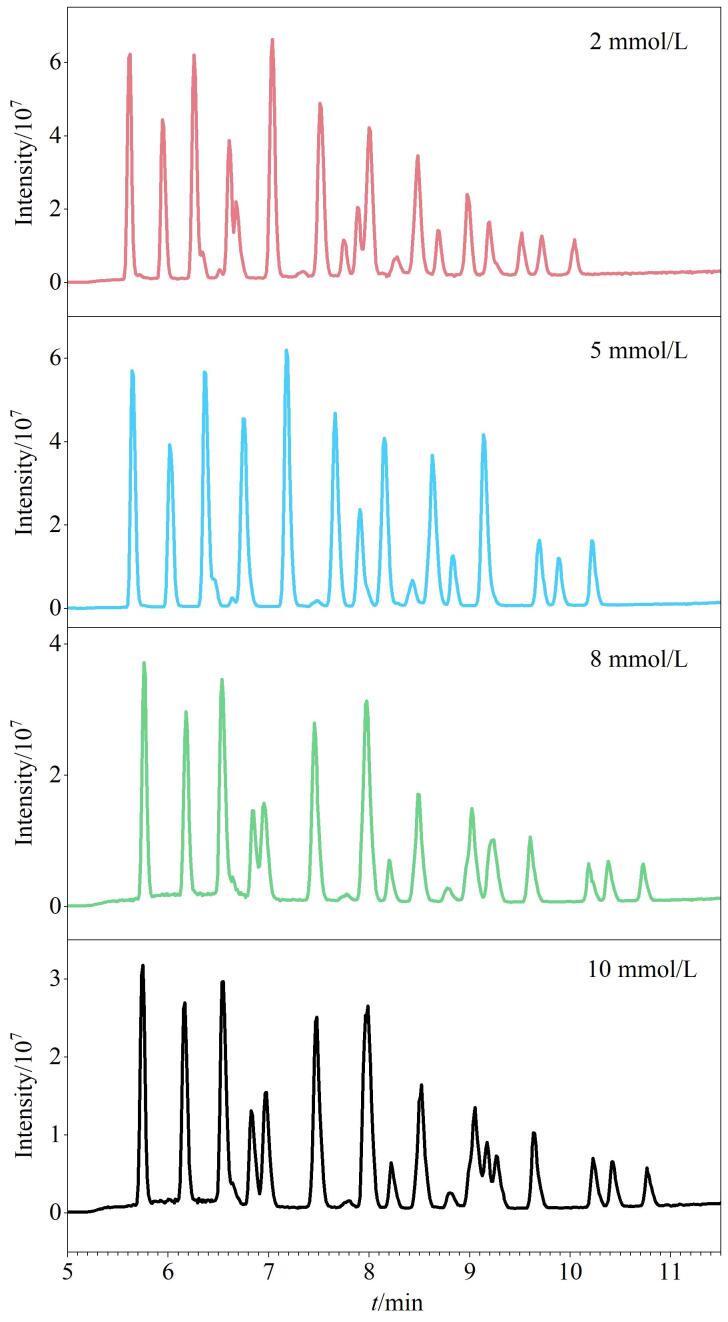
采用不同浓度乙酸铵流动相时31种PFAS的总离子流色谱图

#### 2.1.4 洗脱程序优化

研究文献［^30^］报道，流动相中有机相比例对极性PFAS的灵敏度有非常大的影响。为平衡检测灵敏度与分离选择性，本研究系统优化了梯度洗脱程序，在梯度终点测试90%、95%及99%甲醇比例对灵敏度的影响，并设置3 min平衡时间，确保极性相近化合物实现基线分离。结果发现95%的甲醇在保证目标物响应的同时可实现全谱目标物有效分离且具有良好的峰形（见图3）。

**图3:**
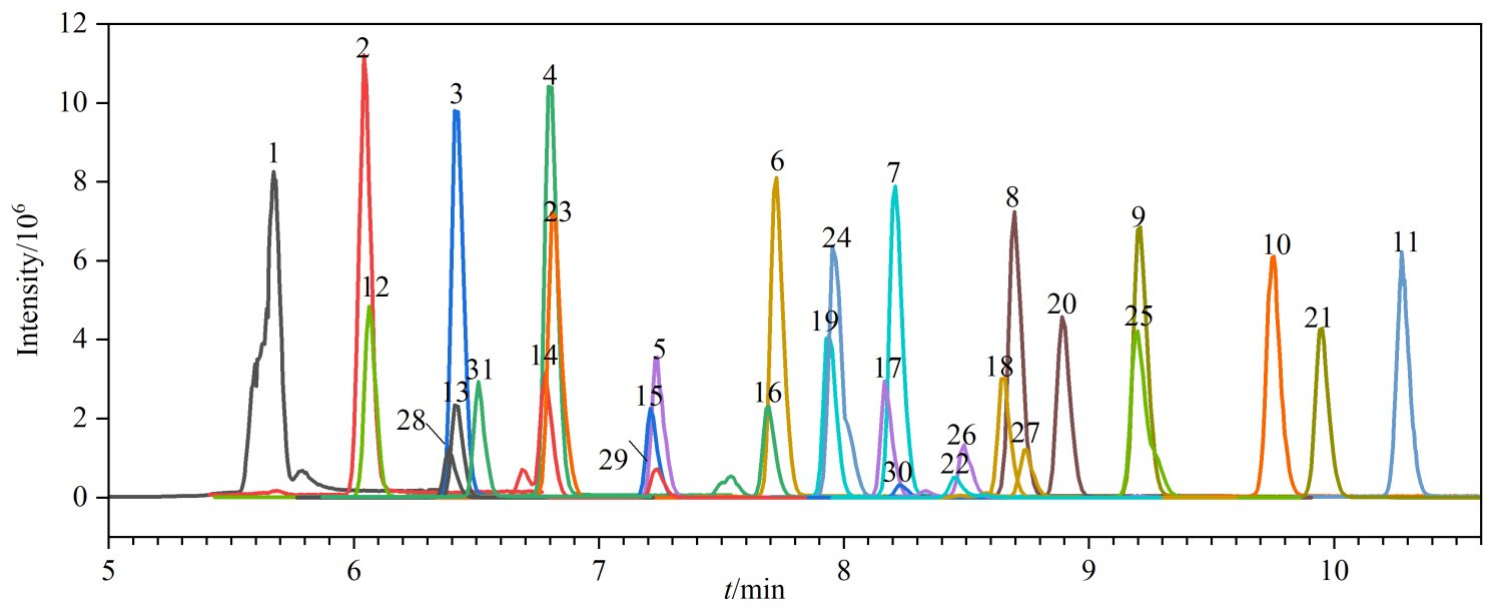
31种PFAS（1 000 ng/L）标准溶液的色谱图

### 2.2 溶剂的选择

研究显示质谱检测时PFAS溶解在不同比例的甲醇-水溶剂中灵敏度和分离度均有差异^［31］^。因此本研究系统考察了4种溶剂（水及甲醇-水混合溶剂（体积比分别为1∶1、5∶1和9∶1））对目标物峰面积和峰形的影响。结果表明，采用纯水为溶剂时，长链PFAS（C≥8，包括PFUnDA、PFDoDA、PFTrDA、PFTeDA、8∶2Cl-PFESA、10∶2Cl-PFESA及FOSA）的峰面积显著低于其余3种含甲醇的混合溶剂体系。甲醇-水混合溶剂体系中甲醇比例的增加并未显著提升目标PFAS的峰面积，各溶剂比例下色谱峰均呈现良好的对称性，峰形平滑无明显展宽或分裂现象。考虑到本方法直接进样的样品以水为基质，为避免过高稀释倍数导致灵敏度下降，最终选择体积比为1∶1的甲醇-水为后续分析的溶剂。

### 2.3 进样量优化

进样量需兼顾仪器硬件限制（定量环上限50 µL）与目标物的色谱保留行为。考察1 000 ng/L标准溶液（溶剂为甲醇-水（1∶1））在5~50 µL进样量时峰面积值的变化。结果表明（见图4），随着进样体积增大，峰面积值持续增加，但增速逐渐放缓，可能原因是进样体积增大导致单位时间内进入离子源的绝对溶质量增加，质谱超载，超过最佳离子化效率对应的载量阈值，削弱单位进样量的信号增益。当进样量≥35 µL时，目标物峰形开始畸变，表现为显著前伸，其可能原因是色谱柱超载，造成部分目标物分子未经充分色谱分配而直接穿透；进样量50 µL时前伸现象加剧，积分误差风险显著升高。35 µL进样量下峰面积已达最大值的97%（较50 µL仅低3%），且其峰对称性保持良好。基于灵敏度与色谱峰形完整性的最优平衡，最终选择35 µL作为最佳进样体积。

**图4:**
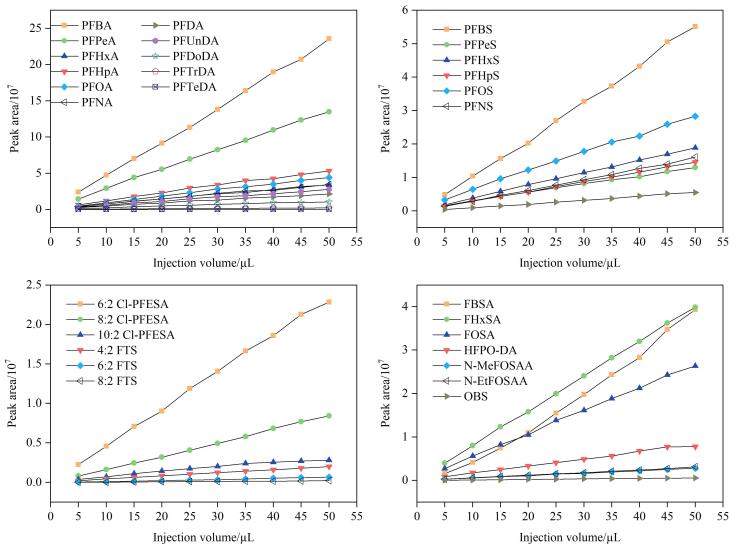
进样体积对目标物峰面积的影响

### 2.4 滤膜的选择

为避免水样中悬浮颗粒物及微生物对分析系统造成堵塞或损伤，样品进样分析前需进行过滤。鉴于PFAS可能吸附于滤膜导致其定量不准确，本研究评估了聚丙烯（PP）、尼龙（nylon）、聚四氟乙烯（PTFE）及聚醚砜（PES）4种不同材质的滤膜对目标物的吸附影响，同时设置空白对照样品（未添加PFAS标准品的溶剂过滤）用于监测试验过程中是否存在滤膜污染。结果显示，所用滤膜均未引入可检出的PFAS污染。将含31种PFAS的混合标准溶液（1 000 ng/L，溶剂为甲醇-水（1∶1，*V/V*））分别通过不同滤膜进行过滤（无预冲洗），通过计算回收率（即过滤后与过滤前峰面积百分比）来评估滤膜造成的吸附损失。结果表明（见图5）：Nylon滤膜对长链（C≥8）及磺酸类PFAS吸附显著，15种PFAS回收率<80%；PTFE滤膜对长链PFCAs（C≥11）吸附严重，PFTrDA和PFTeDA回收率仅为36.5%和9.6%；使用PES滤膜过滤后多数PFAS的回收率>90%，并且9种内标的回收率为74.0%~93.0%，但4∶2 FTS（143.0%）和8∶2 FTS（131.7%）异常偏高，考虑到PES滤膜自身无FTS本底浸出，此异常可能源于滤膜引入的基质效应，其具体机制有待进一步研究。对比而言，使用PP滤膜过滤后31种PFAS和9种内标的回收率均值为100.1%±3.3%（范围87.5%~105.3%），说明PFAS在PP滤膜上吸附较低。因此，综合回收率稳定性和准确性，最终选择PP滤膜对水样进行过滤。

**图5:**
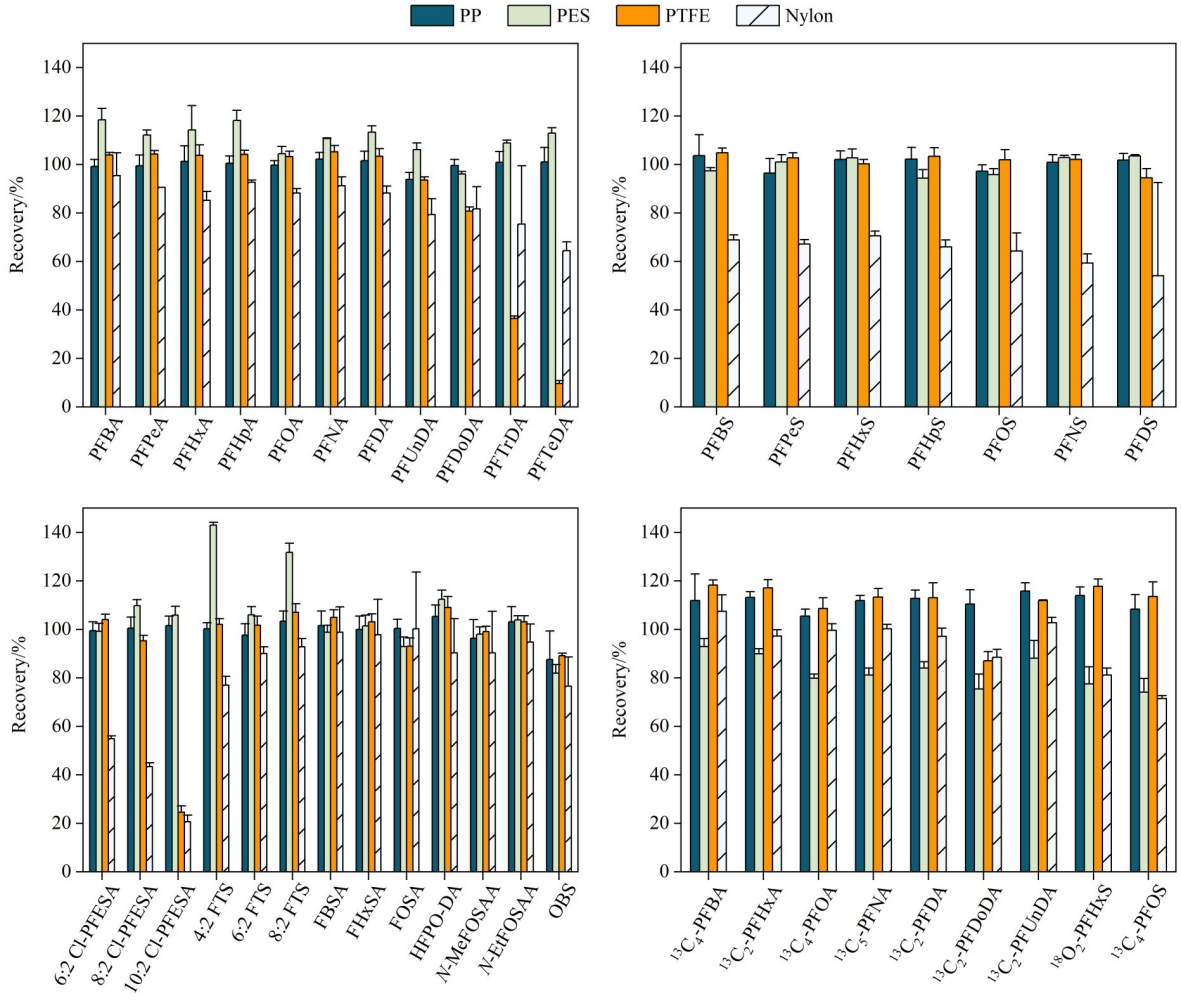
过滤器材质对目标物回收率的影响（*n*=6）

### 2.5 基质效应评估

本研究对前处理方法的基质效应（ME）进行了评估，ME=［（*A*_2_-*A*_1_）/*A*_1_］×100%^［32］^，*A*_1_是溶剂标准溶液中PFAS的峰面积，*A*_2_是同浓度基质匹配校准溶液中PFAS的峰面积。ME>0为增强效应，ME<0为抑制效应。ME在0~±20%范围内可忽略不计，±20%~±50%为中等程度基质效应，>50%或<-50%为较强基质效应。本实验评估20 ng/L和500 ng/L两个加标水平下目标PFAS在地下水样品中的基质效应（见表2）。结果表明，基质效应范围为-27.6%~36.9%，部分目标物呈现中等基质效应。因此，为确保定量准确性，后续采用稳定同位素内标法校正基质效应。

**表 2:** 地下水样品中31种PFAS的基质效应

Analyte	MEs/%		Analyte	MEs/%		Analyte	MEs/%	
	20 ng/L	500 ng/L		20 ng/L	500 ng/L		20 ng/L	500 ng/L
PFBA	-1.6	-27.6	PFBS	21.0	-6.4	FHxSA	20.8	-1.0
PFPeA	7.2	-12.0	PFPeS	19.0	-2.0	FOSA	22.8	1.9
PFHxA	24.9	-4.3	PFHxS	22.4	-1.2	4∶2 FTS	15.3	-5.7
PFHpA	26.3	-0.5	PFHpS	24.5	-1.5	6∶2 FTS	23.7	-1.3
PFOA	36.9	1.1	PFOS	25.8	-1.9	8∶2 FTS	-5.5	-12.8
PFNA	15.0	-1.7	PFNS	24.6	-3.5	HFPO-DA	6.8	-6.0
PFDA	12.6	-9.5	PFDS	16.1	0.6	*N*-MeFOSAA	23.5	-3.1
PFUnDA	22.5	-0.2	6∶2 Cl-PFESA	21.3	-1.7	*N*-EtFOSAA	22.2	-1.9
PFDoDA	13.5	6.9	8∶2 Cl-PFESA	22.2	-2.1	OBS	15.9	-6.9
PFTrDA	12.6	11.7	10∶2 Cl-PFESA	-14.5	2.3			
PFTeDA	-10.5	14.7	FBSA	18.1	-3.8			

### 2.6 方法比较

为量化固相萃取法的富集效能与直接进样法灵敏度的等效关系，基于相同加标水平（2 ng/L）和内标量（2 ng），比较两种方法的回收率。直接进样方法处理流程见1.3节，SPE方法参考文献［^33^］，并系统考察了50、100及200 mL 3种富集体积。结果表明（见图6），直接进样法回收率为66.7%~125.0%。SPE方法在3种富集体积下的整体回收率为56.4%~131.5%。具体而言，多数短链PFAS（C<8，如PFBA、PFPeA、PFHxA、PFHpA、PFNA、PFNS、FBSA、FHxSA）及部分长链PFAS（如PFUnDA、6∶2 FTS、OBS）的回收率在富集体积从50 mL增至200 mL的过程中略有提升，但趋势并不明显；PFSAs（除PFNS外）的回收率无明显变化。上述现象表明，在2 ng/L的加标水平下，当富集体积超过一定限度（如50 mL）后，继续增大体积对多数PFAS的萃取效率提升有限。值得注意的是，部分长链PFAS（如PFDoDA、PFTrDA、*N*-EtFOSAA）的回收率随富集体积增加明显下降，表明此类物质在大体积富集过程中可能存在更高的损失风险。整体而言，直接进样法与富集体积为50 mL的SPE方法回收率水平相当。因其避免了SPE过程中的吸附与操作损失，表现出良好的回收率稳定性。因此，在常规PFAS检测中，直接进样法可作为SPE前处理的有效替代，在满足灵敏度要求的同时显著提高分析效率。

**图6:**
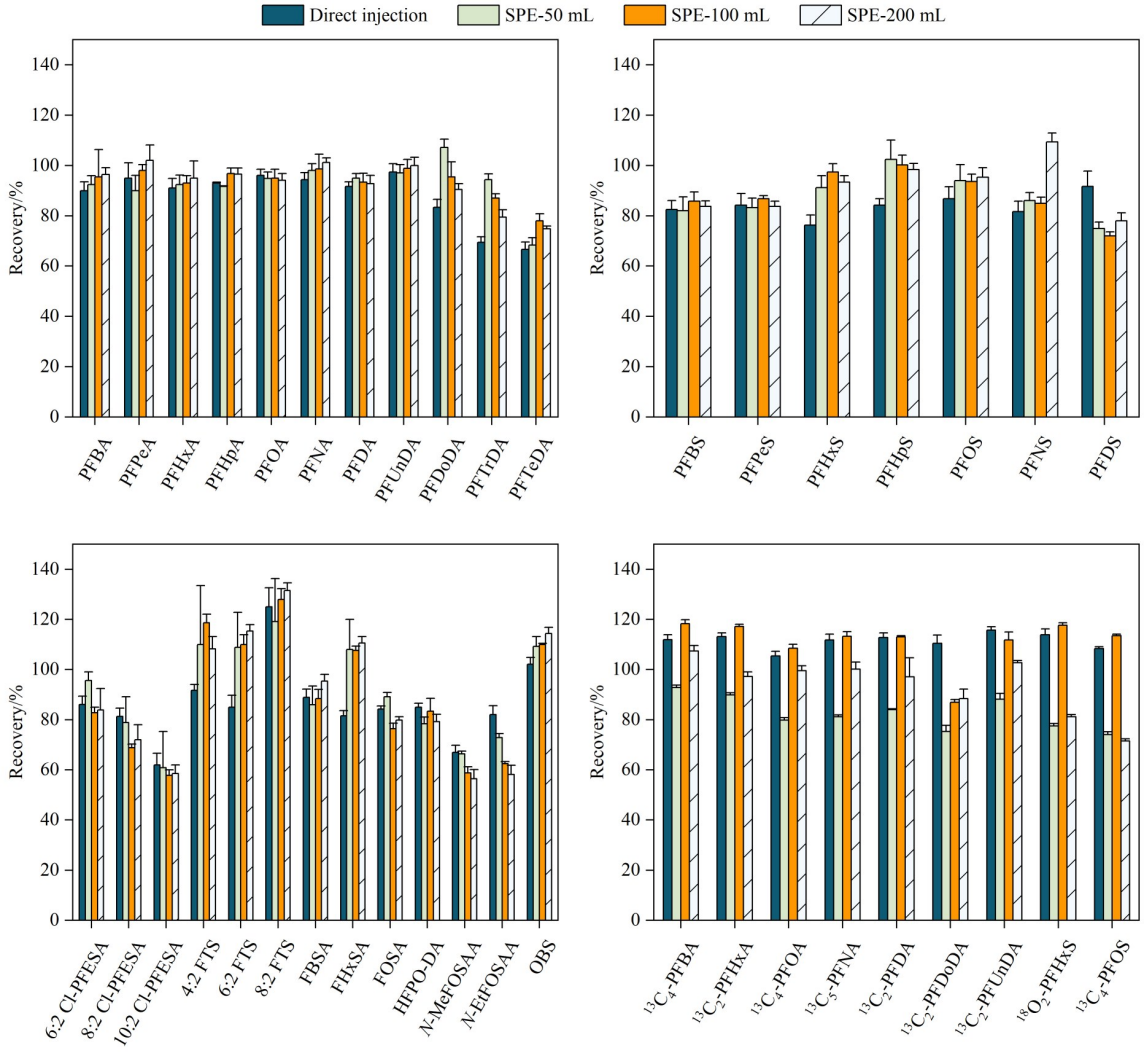
直接进样与固相萃取法对目标物回收率的影响（*n*=3）

### 2.7 线性范围与检出限

本研究采用内标法定量，标准曲线系列质量浓度为1、2、5、10、20、50、100、200、500、1 000 ng/L，按照1.4节的仪器条件检测，以各PFAS与对应内标的质量浓度比为横坐标（*x*），峰面积比为纵坐标（*y*）绘制标准曲线，使用1/*x* ^2^加权回归，线性相关系数*R* ^2^均大于0.994。方法的检出限（LOD）和定量限（LOQ）分别由3倍和10倍的信噪比（*S/N*）计算得出，其范围分别为0.007 1~3.0 ng/L和0.024~10 ng/L，详见表3。

**表 3:** 31种PFAS的线性范围、相关系数、检出限、定量限和加标回收率（*n*=6）

Analyte	Linear range/（ng/L）	*R* ^2^	LOD/ （ng/L）	LOQ/ （ng/L）	2 ng/L		10 ng/L		500 ng/L	
					Recovery/%	RSD/%	Recovery/%	RSD/%	Recovery/%	RSD/%
PFBA	10-1000	0.996	3.0	10	107.7	2.9	103.0	13.6	101.4	7.8
PFPeA	10-1000	0.997	2.1	7.1	130.2	2.1	109.1	2.3	96.0	16.5
PFHxA	1-1000	0.994	0.076	0.25	110.6	1.5	110.7	2.8	102.5	15.5
PFHpA	1-1000	0.998	0.15	0.50	118.3	0.4	105.6	3.4	112.2	5.6
PFOA	1-1000	0.998	0.18	0.61	104.9	2.2	106.3	2.9	102.7	4.8
PFNA	1-1000	0.995	0.10	0.35	108.3	4.2	108.8	0.7	101.6	0.9
PFDA	1-1000	0.994	0.098	0.33	107.2	8.4	107.5	3.4	101.4	2.7
PFUnDA	1-1000	0.995	0.23	0.78	108.5	2.1	107.5	12.3	100.9	1.4
PFDoDA	1-1000	0.996	0.043	0.14	104.4	0.6	97.0	10.3	97.0	3.3
PFTrDA	1-1000	0.997	0.16	0.55	86.0	1.4	90.2	15.6	90.6	2.8
PFTeDA	1-1000	0.997	0.22	0.74	70.6	5.3	77.9	5.8	79.3	3.6
PFBS	1-1000	0.999	0.092	0.31	97.8	1.9	97.2	4.2	97.9	7.5
PFPeS	1-1000	0.999	0.052	0.17	105.6	3.3	99.5	3.5	104.0	6.4
PFHxS	1-1000	0.999	0.10	0.33	106.3	3.1	101.6	6.5	102.0	2.6
PFHpS	1-1000	0.999	0.076	0.25	98.8	4.1	96.1	4.6	100.7	0.3
PFOS	1-1000	0.999	0.064	0.22	103.3	0.6	98.2	16.4	99.1	1.6
PFNS	1-1000	0.999	0.061	0.20	98.7	3.0	94.4	10.6	96.0	1.5
PFDS	1-1000	0.999	0.055	0.18	97.2	2.9	94.2	10.4	95.8	2.8
6∶2 Cl-PFESA	1-1000	0.997	0.027	0.090	99.5	4.5	96.1	18.6	97.3	11.5
8∶2 Cl-PFESA	1-1000	0.995	0.017	0.055	99.1	5.8	100.5	7.9	100.9	4.5
10∶2 Cl-PFESA	1-1000	0.998	0.012	0.042	67.2	7.1	82.8	6.8	86.9	6.7
FBSA	1-1000	0.999	0.010	0.034	101.7	3.3	96.1	3.0	99.6	0.8
FHxSA	1-1000	0.999	0.0071	0.024	94.0	2.9	90.2	5.2	93.6	3.5
FOSA	1-1000	0.999	0.011	0.036	102.7	1.4	95.1	1.5	96.6	2.2
4∶2 FTS	1-1000	0.998	0.028	0.095	112.2	1.2	108.1	9.6	109.5	15.5
6∶2 FTS	1-1000	0.999	0.024	0.079	100.7	2.0	96.5	7.0	99.5	16.3
8∶2 FTS	1-1000	0.999	0.079	0.26	106.0	0.9	93.9	18.4	91.4	0.7
HFPO-DA	2-1000	0.996	0.49	1.6	96.3	1.5	94.6	11.2	97.6	1.2
*N*-MeFOSAA	1-1000	0.999	0.11	0.36	106.7	0.4	102.1	5.9	100.3	3.5
*N*-EtFOSAA	1-1000	0.996	0.056	0.19	106.4	2.2	100.2	5.3	104.3	7.6
OBS	1-1000	0.999	0.060	0.20	93.3	4.0	85.1	6.2	81.0	8.4

### 2.8 回收率

为进一步确认方法的有效性和精密度，以含有较低浓度PFAS的地下水样本为空白基质，分别添加2、10和500 ng/L的混合标准溶液，按照优化后的方法进行检测，每个水平平行测定6次，计算加标回收率和定量结果的相对标准偏差（RSD）。31种PFAS的加标回收率为67.2%~130.2%，RSD为0.30%~18%。表明该方法具备可靠的准确性与精密度。

### 2.9 实际样品的分析

将本方法用于地下水样品中PFAS的定量分析，结果见表4。

**表 4:** 地下水样品中PFAS的含量

PFAS	Contents/（ng/L）									
	No. 1	No. 2	No. 3	No. 4	No. 5	No. 6	No. 7	No. 8	No. 9	No. 10
PFBA	13.40	7.60	46.00	86.60	2.60	5.80	51.80	147.40	34.20	43.80
PFPeA	4.40	8.80	16.40	16.60	3.40	25.00	31.80	49.80	23.00	33.00
PFHxA	9.00	7.20	29.20	10.80	1.60	35.20	48.80	73.80	22.20	36.60
PFHpA	7.80	8.60	36.00	8.20	0.60	26.00	62.40	30.60	24.80	19.20
PFOA	120.60	18.60	155.20	11.60	9.20	112.40	246.20	21.00	71.20	30.00
PFNA	3.40	3.60	2.40	0.20	1.00	15.80	30.60	0.40	2.80	ND
PFDA	1.00	2.00	ND	ND	ND	5.60	6.20	ND	ND	ND
PFUnDA	ND	ND	ND	ND	ND	0.40	ND	ND	ND	ND
PFDoDA	ND	ND	1.00	0.40	ND	ND	ND	ND	ND	ND
PFBS	1.60	2.00	17.00	55.00	0.40	4.80	8.00	11.60	9.00	5.00
PFPeS	ND	ND	ND	ND	ND	2.00	1.20	ND	ND	ND
PFHxS	6.60	ND	0.20	ND	ND	ND	0.20	ND	ND	ND
PFHpS	ND	0.20	ND	ND	ND	ND	0.20	ND	ND	ND
PFOS	1.00	96.60	ND	ND	0.20	18.80	11.40	0.20	ND	ND
6∶2 Cl-PFESA	ND	7.80	ND	ND	ND	0.80	1.60	ND	ND	ND
FBSA	ND	ND	0.20	ND	ND	ND	3.20	0.80	0.60	ND
FHxSA	ND	ND	ND	ND	ND	ND	0.20	ND	ND	ND
FOSA	ND	ND	ND	ND	ND	0.60	ND	ND	ND	ND
6∶2 FTS	ND	4.40	0.60	0.20	0.40	2.00	1.60	1.00	7.80	0.60
8∶2 FTS	ND	ND	ND	ND	ND	ND	0.40	ND	ND	ND
HFPO-DA	6.40	4.60	9.00	0.80	0.20	2.40	8.20	0.80	11.80	30.60
*N*-MeFOSAA	ND	ND	ND	ND	ND	ND	0.60	ND	ND	ND
*N*-EtFOSAA	ND	ND	ND	ND	ND	ND	2.20	ND	ND	ND
OBS	1.20	1.40	1.00	1.00	1.00	2.40	4.20	1.00	1.20	1.00

ND： not detected.

由表4可以看出，地下水中共检出24种PFAS，总含量为20.6~521 ng/L，PFCAs占总含量的84.2%，其中PFOA（11.6~246.2 ng/L）与PFBA（2.6~147.4 ng/L）为主要污染物，其次是PFBS（0.4~55.0 ng/L）和PFOS（ND~96.6 ng/L）。其余7种目标PFAS（C>9）均未检出。

## 3 结论

研究建立了直接进样定量分析地下水中31种PFAS的UPLC-MS/MS检测方法。方法采用同位素内标法，有效克服了地下水基质中无机离子与溶解性有机物的干扰，定量限达ng/L级。相较于固相萃取法，本方法显著简化了前处理流程，提高了工作效率，具有简单、快速、精密度高、灵敏度高、样品消耗量小的优点，为地下水PFAS污染监测提供了可靠的技术方案。所建立的方法体系在进一步验证的基础上，具备拓展应用于地表水及污水处理厂污水等复杂环境水体的潜力。

尽管本研究建立的方法在快速筛查方面优势显著，但由于缺乏净化与浓缩富集步骤，存在一定的局限性。未来应在以下方面进一步深化研究：其一，在面对成分复杂的环境样品时，方法的抗干扰能力还需进一步验证优化，可通过优化色谱分离或引入其他净化方法予以增强；其二，对于某些超痕量毒理学关注物质，可尝试耦合在线富集技术或应用灵敏度更高的质谱技术，以突破现有检出限；其三，可与高分辨率质谱联用，从可疑物质筛查向未知PFAS的结构鉴定拓展，从而提升对新污染物的识别能力。总之，本研究不仅提供了一种水体中PFAS的高效筛查方案，更为该技术路线的持续发展奠定了坚实基础。
